# Species composition and community structure of small pest rodents (Muridae) in cultivated and fallow fields in maize‐growing areas in Mayuge district, Eastern Uganda

**DOI:** 10.1002/ece3.5371

**Published:** 2019-06-20

**Authors:** Alex Mayamba, Robert M. Byamungu, Rhodes H. Makundi, Didas N. Kimaro, Moses Isabirye, Apia W. Massawe, David Kifumba, Alice Nakiyemba, Herwig Leirs, Mshaka E. Mdangi, Brian E. Isabirye, Loth S. Mulungu

**Affiliations:** ^1^ Faculty of Natural Resources and Environment Busitema University Tororo Uganda; ^2^ Department of Wildlife Management Sokoine University of Agriculture Morogoro Tanzania; ^3^ Pest Management Centre Sokoine University of Agriculture Morogoro Tanzania; ^4^ Department of Engineering Sciences and Technology Sokoine University of Agriculture Morogoro Tanzania; ^5^ Evolutionary Ecology Group Universiteit Antwerpen Antwerpen Belgium; ^6^ Ministry of Agriculture Training Institute (MATI) Ilonga Kilosa Tanzania; ^7^ International Center of Insect Physiology and Ecology (ICIPE) Nairobi Kenya

**Keywords:** community structure, composition, cultivated fields, fallow land, richness, rodents, species diversity

## Abstract

1. Pest rodents remain key biotic constraints to cereal crops production in the East African region where they occur, especially in seasons of outbreaks. Despite that, Uganda has scant information on rodents as crop pests to guide effective management strategies.
2. A capture–mark–recapture (CMR) technique was employed to study the ecology of small rodents, specifically to establish the species composition and community structure in a maize‐based agro ecosystem. Trapping of small rodents was conducted in permanent fallow land and cultivated fields, with each category replicated twice making four study grids. At each field, a 60 × 60 m grid was measured and marked with permanent trapping points spaced at 10 × 10 m, making a total of 49 trapping points/grids. Trapping was conducted monthly at 4‐week interval for three consecutive days for two and half years using Sherman live traps.
3. Eleven identified small rodent species and one insectivorous small mammal were recorded with *Mastomys natalensis* being the most dominant species (over 60.7%). Other species were *Mus triton* (16.1%), *Aethomys hendei* (6.7%), *Lemniscomys zebra* (5.2%), *Lophuromys sikapusi* (4.8%), *Arvicanthis niloticus* (0.9%), *Gerbilliscus kempi* (0.1%), *Graphiurus murinus* (0.1%), *Steatomys parvus* (0.1%), *Dasymys incomtus* (0.1%), and *Grammomys dolichurus* (0.1%). Spatially, species richness differed significantly (*p* = 0.0001) between the studied field habitats with significantly higher richness in fallow land compared with cultivated fields.
4. Temporally, total species richness and abundance showed a significant interaction effect over the months, years, and fields of trapping with significantly (*p* = 0.001) higher abundances during months of wet seasons and in the first and third year of trapping. In terms of community structure, higher species diversity associated more with fallow field habitats but also with certain rare species found only in cultivated fields.
5. Synthesis and applications. Based on these findings, management strategies can be designed to target the key pest species and the most vulnerable habitats thus reducing the impact they can inflict on field crops.

## 1 INTRODUCTION

Rodents exhibit irregular population dynamics with occasional outbreaks, typically occurring over extensive areas (Fiedler, 1988; Leirs, Verhagen, Verheyen, Mwanjabe, & Mbise, 1996). Globally, they are among the most destructive vertebrate pests to cereal crops (Leirs, 2003; Singleton, Hinds, Leirs, & Zhang, 1999; Stenseth et al., 2003), with profound crop damage impact in the low developing countries in Africa (Mdangi et al., 2013; Makundi, Oguge, & Mwanjabe, 1999), Asia (Singleton, 2003), and Indonesia (Geddes, 1992). Particularly, studies in the East African region (Leirs, Singleton, & Hinds, 1999; Makundi et al., 1999; Mulungu, 2003; Mwanjabe, 1990) have identified several rodent species that are important and responsible for crop yield loss and in lowering of crop qualities. In this region, rodents commonly cause 5%–15% damage on maize crop (Mwanjabe & Leirs, 1997), but projections indicate that it can reach over 80% in seasons of outbreaks (Mulungu, 2003). Largely, multimammate rats (*Mastomys natalensis*) are pointed out as the most important rodent pests involved in crop damage in the sub‐Saharan Africa (Fiedler, 1988) though other groups such as *Gerbiliscus* spp. and *Arvicanthis* spp. are also involved (Makundi et al., 1999). These rodent groups are known for their damages on a diversity of cereal crops with preponderant impact on maize and rice, the crops which are important in food security across the East African region.

In Uganda, cereal crops form a key component of the crop production sector and contribute significantly to the dietary diversity of many rural and urban communities (Shellemiah & Rubaihayo, 2013). However, production of diverse cereals is still low due to several production constraints including massive loss due to rodent pest damages (Nabbumba & Bahiigwa, 2003; Waddington, Li, Dixon, Hyman, & Vicente, 2010). Currently, rodent management strategies in the country are minimal due to the scant information available on rodents as pests to guide management (Eisen et al., 2013; Moore et al., 2015). Specifically, knowledge on the species composition and community structure is known fundamental facts for a successful and acceptable pest control strategy (Hoare & Hare, 2006; Parsons, Banks, Deutsch, Robert, & Munshi‐South, 2017; Simberloff, 2014). Presently, literature available in the country focuses on rodents as potential disease vectors to human and livestock (Amatre et al., 2009; Bochert et al., 2010; Eisen et al., 2010) but less so as crop pests. No detailed studies exist in the country on rodents as field crop pests, and little is known about rodent communities in agriculture cropping systems. This study thus aimed at determining the species composition and community structure of small pest rodents in cultivated and fallow land fields in maize‐growing areas in Eastern Uganda, a step toward developing a successful pest management strategy in the country. The knowledge on rodent diversity of rodents and their distribution in the environment will enable design of appropriate management strategies that will target harmful species while sparing the beneficial ones (Singleton, Sudarmaji, Jacob, & Krebs, 2005).

## 2 MATERIALS AND METHODS

### 2.1 Study site

**Figure 1:**
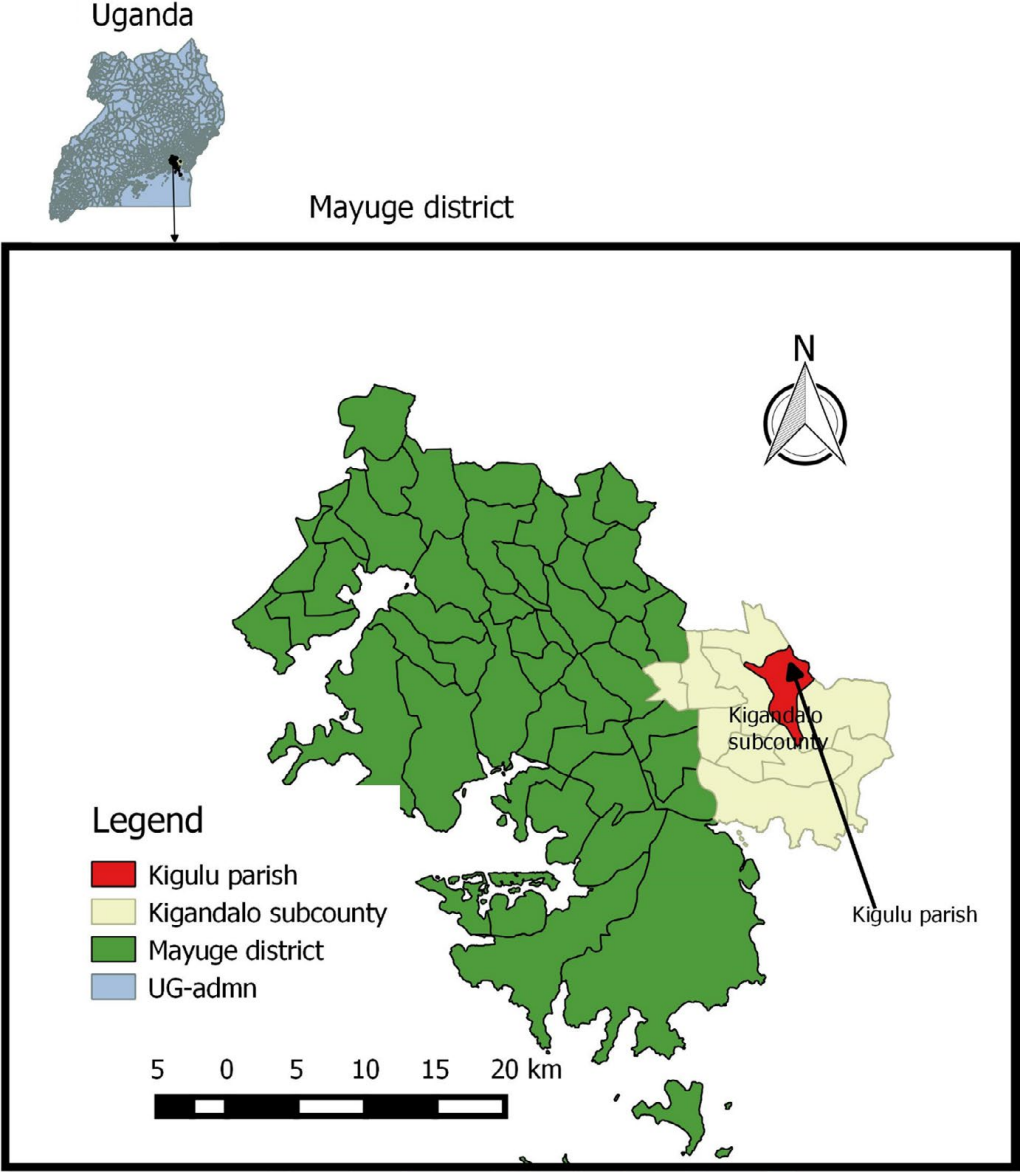
Map showing the location of the study site, Kigandalo subcounty, Mayuge district Eastern Uganda

The study was conducted in Kigulu parish, Kigandalo subcounty, Mayuge district in Eastern Uganda (06°16′S, 37°31′E), ~1,020 m above sea level (Figure 1). The study area experiences a bimodal rainfall pattern, characteristic of Eastern Uganda in the Lake Victoria Crescents agro‐ecological zone. There are two rainy seasons in the year: first rainy season normally occurs between March and end of May with a short dry period (June–August). The second rainy season occurs between August and end of November, then a dry spell from December to February of the following year. Due to the intense demand for agricultural and pasture land in this region, land is highly fragmented and natural forests are very scarce and in small patches.

### 2.2 Sampling procedure

Permanent trapping fields for the experiment were obtained through negotiation with landowners and agreements formally made. A purpose sampling technique was employed, where experimental fields where selected basing on certain criteria; availability of the required plot size (60 x 60m), acceptablity of the land owner to offer the area for a period of two years and this targeted both cultivated field and fallow land habitats. In this area, land use is highly fragmented, and thus, we targeted fields that could measure about 70 m × 70 m and the permanent trapping grids were measured off starting at 10 m from the boundary line. In each of the two habitat types, two replicate grids were obtained making a total of four trapping fields at a minimum distance of 500 m from each other. At each of the identified field sites, a 60 m × 60 m grid was marked and permanent trapping points set. The fallow land fields were initially dominated with heavy thick patches of tick berry (*Lantana camara*) but were subsequently reduced due to animal grazing. Other weedy species noted were perennial and annual grasses (Gramineae) of several species, which are common in disturbed soils and uncultivated fallow lands. They included guinea grass (*Panicum maximuma*) couch grass (*Digitaria scalarum*), black jack (*Bidens pilosa*), star grass (*Cynodon dactylon), and* wandering jew (*Commellina bengelensis*) among others. The fallow lands were surrounded by cultivated fields, which, during the wet season, were planted with maize, beans, cassava, and sweet potatoes. After crop harvest, these fields were left with standing stubble and often slash and ox‐plow were the main land preparation methods before the next wet/planting season started.

Cultivated fields were planted with maize intercropped with beans in the first year of the study (2016), but in the subsequent seasons, cassava was introduced as a way of crop rotation due to the parasitic witch weed (*Striga* sp.) in the area, which deprives the maize crop from water and other mineral nutrients. Other commonly encountered weeds in the cultivated fields included star grass (*Cynodon dactylon*), couch grass (*Digitaria scalarum*), black jack (*Bidens pilosa*), guinea grass, and wandering jew (*Commellina bengelensis*). Fragments of mixed crop gardens comprising of coffee, beans, bananas, sweet potato, and cassava also surrounded these cultivated study fields.

### 2.3 Trapping procedure

Using Sherman live traps (H.B. Sherman Traps, Inc.) a capture–mark–release trapping technique was applied following the procedure described in Aplin, Brown, Jacob, Krebs, and Singleton (2003). For each trapping grid, 49 Sherman live traps were set in a 60 m × 60 m configuration (seven trapping lines with seven trapping stations, 10 m apart). Trapping was conducted monthly at 4‐week intervals. A single Sherman trap baited with peanut butter mixed with maize flour was placed at each trapping station for three consecutive days. Traps were inspected every morning during the three days, and captured animals were checked for sexual maturity status, weighed, toe clip coded, and released at the points of capture. Both traps with and without animals were rebaited with fresh bait for the following day trapping. The study lasted for two and half years from January 2016 to May 2018. The nomenclature by Wilson and Reeder (2005) was used as the main reference to identify the rodent species captured in the study areas. The community structure in this study was described as relative composition based on the trappable rodent species in the study sites. The proportional species composition was presented as percentage based on the relative abundance of each species over the study period. The density of animals per/0.5 ha was estimated for each three‐day trapping session using the M(h) estimator of the program CAPTURE for a closed population, which allows for individual variations in trapping probability (White, Anderson, Burnham, & Otis, 1982) and is the most commonly used test in other studies thus allows better comparison with those studies.

### 2.4 Data processing and analysis

Data from the four grids were pooled and formed two data sets: cultivated field and fallow land field to obtain total small rodent diversity per habitat. Species richness and abundance were calculated using the pooled data for cultivated fields and fallow land fields. All variables were tested for normality using Shapiro–Wilk test, and the strongly skewed variables were transformed prior to analyses whether necessary, to meet the assumption of normality and homogeneity of variances (Wilcoxon, 1945). Paleontological Statistics software (PAST; Hammer et al., 2002) was used to calculate diversity measures: species richness, Simpson Diversity Index, evenness, and dominance. Species accumulation curves and rank abundance curves were obtained for the two field categories using R software Vegan package (R software version 3.3.2; R Core Team, 2013). The monthly differences in small rodent richness and abundance between cultivated and fallow habitats were tested with analysis of variance (ANOVA) in XLSTAT (XLSTAT, 2017). Where the ANOVA test indicated significant differences, post hoc Tukey (HSD) test was used. Richness was used as a measure of the number of species in the two field habitats. Species diversity estimations were made by the Simpson's Diversity Index to consider both the richness and evenness. The index was calculated using the formula:

$$D=1-\frac{\sum n(n-1)}{N(N-1)}D=1-\frac{\sum n(n-1)}{N(N-1)}D=1-\frac{\sum n(n-1)}{N(N-1)}$$

where *D*, Simpson diversity (D′); *n* = number of individuals of each species, and *N* = total number of individuals of all species.

A *t* test was used to compare the Simpson's Diversity Indices between trapping grids.

Species turnover was computed to determine the rate of species change in time and space; temporal turnover (β_T_) in species richness between years was calculated for each site as the total number of species found within that site (over the two and half years) minus the mean number of species per year for that site (**α**). Spatial turnover (β_S_) was calculated as the total number of species found within a habitat type (over the two and half years) minus the mean number of species per site for that habitat type (over the two and half years).

The Bray–Curtis similarity index (Hammer et al., 2002) was used to compare similarities among zones and to construct a species composition similarity dendrogram for the three zones. The nonmetric multidimensional scaling ordination was used to plot species association with habitat type.

## 3 RESULTS

### 3.1 Small mammal species composition

**Figure 2:**
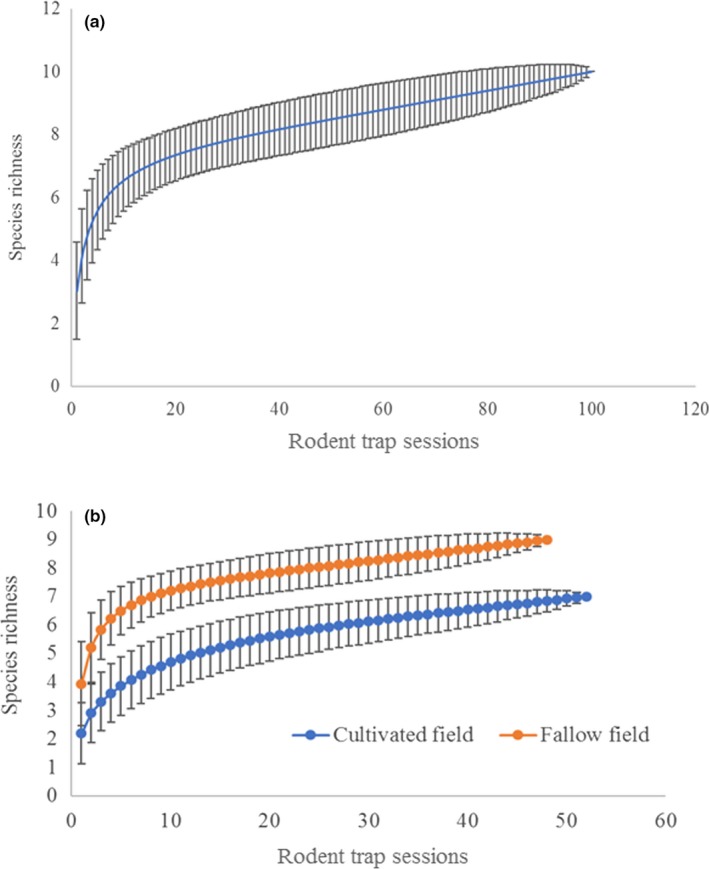
Species accumulation for all samples (a) and (b) for the separate studied fields (Fallow and cultivated fields with ± Standard deviation)

Out of the 17,052 trap nights made, 1,061 and 1,355 small mammal individuals were trapped in cultivated and fallow land fields, respectively. These comprised of 11 small rodent species and one insectivorous small mammal species making a total of 12 small mammals (Table 1). Multimammate rat (*Mastomys natalensis*) was the most abundant rodent species with 727 (68.5%) individuals in cultivated fields and 740 (54.6%) individuals in fallow land fields, while the least was *Gerbilisicus kempi, Gramommys dolichurus*, *Dasmys incomtus,* and *Steatomys parvus*. The former four rodent species were very scarce as only one individual each was captured for the whole study period (Table 1). The results also showed that fallow fields were species richer (10 small rodent species) compared with cultivated fields (nine small rodent species; Table 1). The species accumulation curve plotted (Figure 2a) showed a good sampling effort as it tended to level off after the 20th trapping session, with minimal encounters of new species after, but also indicates that a few more species can be trapped with more years of trapping. Additionally, separate curves for the habitats were plotted and fallow fields displayed a slightly higher accumulation curve compared with cultivated fields (Figure 2b), implying a higher probability of encountering more species in fallow field habitat with sampling. The overall maximum species estimated by Chao 2, Jackknife 1, and Bootstrap richness estimators in the study area for the two and half years of the study was 13 species. Simpson species diversity index showed relatively higher diversity for fallow field (0.617) compared with cultivated field (0.467) but was not significantly different (*p* > 0.05). Species evenness was higher in fallow field (42.04%) compared with cultivated field (34.17%).

**Table 1:** Inventory of small rodent species and an insectivorous mammal recovered during the study period in the cultivated and fallow field habitats in Mayuge district, Eastern Uganda, year 2016–2018

Species	Total number of individuals (% contribution) in Cultivated field	Total number of individuals (% contribution) in Fallow field	Over all number (% contribution)
Small rodent species			
1. *Mastomys natalensis*	727 (68.5)	740 (54.6)	1,467 (60.7)
2. *Mus triton*	210 (19.8)	180 (13.3)	390 (16.1)
3*. Aethomys hendei*	35 (3.3)	128 (9.4)	163 (6.7)
4. *Lemniscomys zebra*	15 (1.4)	102 (7.5)	117 (4.8)
5. *Lophuromys sikapusi*	6 (0.6)	67 (4.8)	73 (3.3)
6. *Arvicanthis niloticus*	1	25	26
**7.** *Graphiurus murinus*	0	15	15
8. *Gerbilliscus kempi*	1	0	1
9. *Gramommys dolichurus*	0	1	1
10*. Steatomys parvus*	0	1	1
11*. Dasmys incomtus*	1	0	1
Insectivorous species			
1. *Crocidura* spp.	65 (6.1)	80 (5.9)	145 (6.0)
Total captured	1,061 (100)	1,352 (100)	2,413 (100)
Total trap nights	8,820	8,232	17,052
Species richness	9	10	12
Simpson's Diversity Index	0.467	0.617	

**Figure 3:**
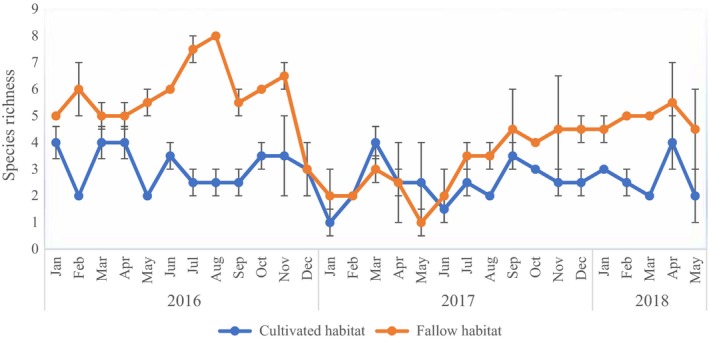
Mean (±*SE*) monthly species richness over the two and half year's study period in fallow and cultivated fields in Mayuge district, Eastern Uganda

In terms of temporal variations in species richness and abundance, there was a significant (*F*_28,29_ = 2.819, *p* = 0.004) interaction effect between months and years of the study for richness within fallow land habitat. Significantly, more species were observed in the first year of trapping (2016) in June, July, and August and then November (Figure 3). Lowest species recovery was noted to have occurred in the second year of trapping (2017), specifically in the month of May (Figure 3). Within cultivated field habitat, there was also significant interaction effect between months and years of the study for species richness (*F*_28,29_ = 1.857, *p* = 0.054). Significantly, fewer species recovery was observed in the second year of trapping, in January, May, and June which differed from the rest. Generally, there was almost consistence in the number of species recovered monthly over the study period (Figure 3).

**Figure 4:**
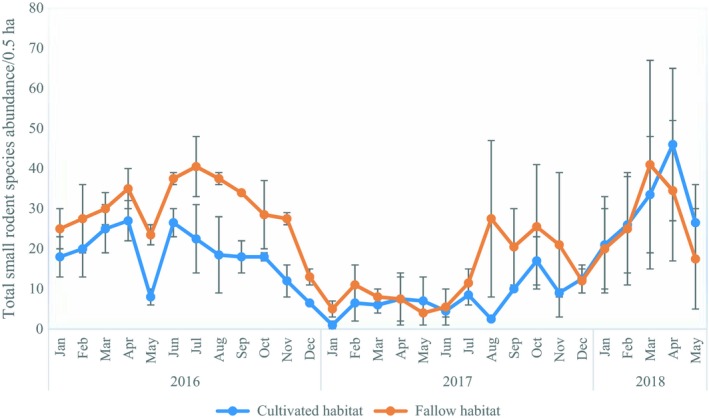
Mean (±*SE*) monthly small rodent abundance over the two and half year's study period in fallow and cultivated field habitats in Mayuge district, Eastern Uganda

The interaction effect between years and months on total small rodent species abundance also showed a significant effect for fallow land (*F*_28,29_ = 2.334, *p* = 0.001). Significantly, higher abundances were obtained in the months of June (38 ± 2/0.5 ha), July (41 ± 8/0.5 ha), August (38 ± 2/0.5 ha) for 2016, and March (41 ± 26/0.5 ha) in 2018. In cultivated field habitats, the interaction effect of year and month of trapping on small rodent abundance was also significant (*F*_28,29_ = 2.612, *p* = 0.007). Significantly, higher abundance was recorded in the last year of trapping (2018) in the month of April (46 ± 19/0.5 ha; Figure 4). Generally, there a was synchrony in temporal changes in rodent abundance over the years in the studied field habitats, with higher abundance in the first year of trapping, then a decline in year two and a steady rise in the third year of trapping.

**Figure 5:**
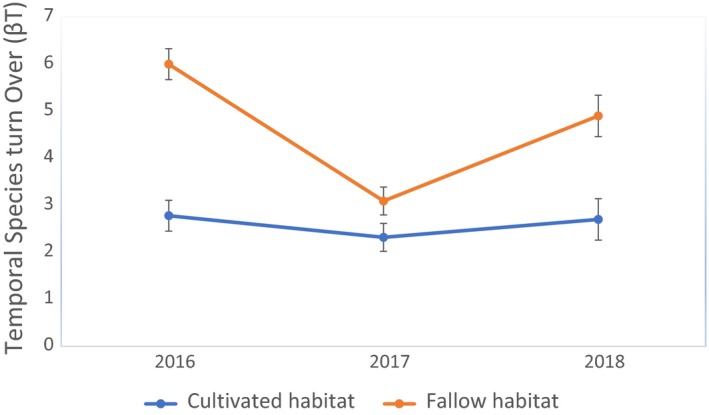
Mean (±*SE*) temporal species turn over (β_T_) for the different years of study in Mayuge district, Eastern Uganda

In terms of species turnover, spatially there was a significant difference (*F*_1,6_ = 9, *p* = 0.024) for the studied field habitats. Fallow field habitats showed significantly higher species turnover (6 ± 1) species compared with cultivated field habitat (4 ± 1). Temporal species turn over (β_T_) also showed a significant difference (*F*_5,44_ = 18.819, *p* = 0.0001) over the three years of the study. The first year of trapping showed a higher species turn over followed by a decline in the second year of trapping and then a rise in the third year of trapping in both habitats (Figure 5).

### 3.2 Small rodent community structure across field trapping habitats

**Figure 6:**
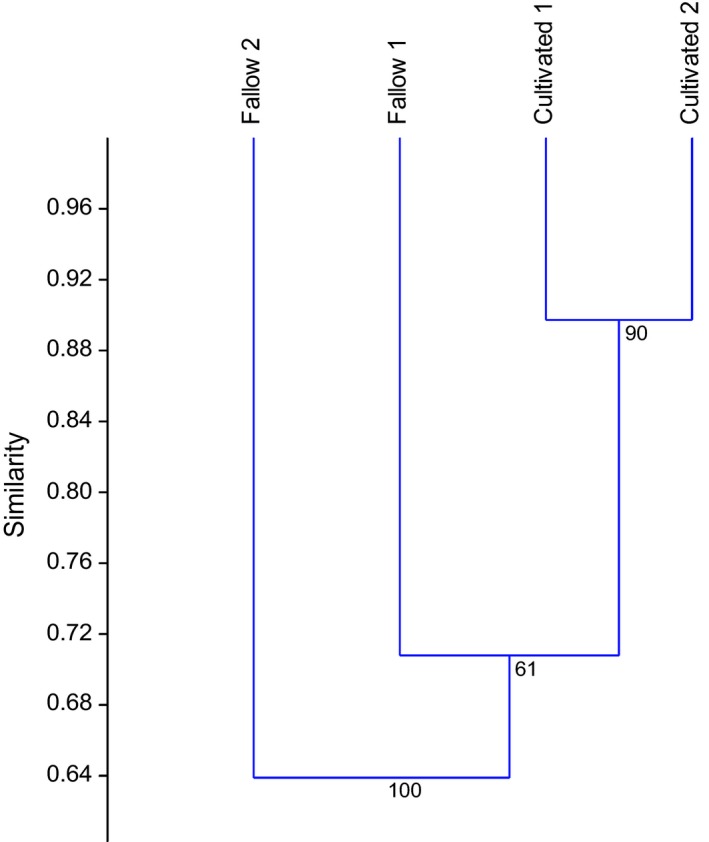
Bray–Curtis similarities in rodent composition among the trapping habitats and species communities in the study

**Figure 7:**
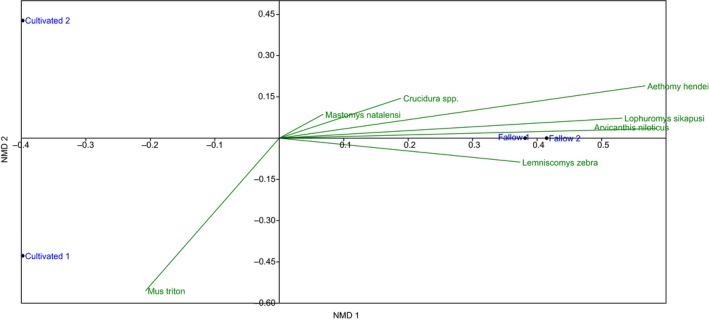
Ordination plots for nonmetric dimensional scaling (NMDS) in rodent community composition among trapping habitats

The trapping habitats were generally similar in composition with respect to rodent species. The Bray–Curtis similarity index generated three clusters—one for the cultivated fields, then separate clusters for fallow fields, with an overall cophenation correlation or cluster accuracy of 97.97% (Figure 6). A nonmetric multidimensional scaling analysis was conducted, and ordination plots were generated with a correlation method. Rodent communities were very distinct between habitats. Some species associated only with certain communities such as *G. kempi* sp. and *D*. *incomtus,* these only associated with cultivated habitats. The ordination plots also revealed that several of the recorded rodent species in the study associated more with fallow habitats. There was one rodent species, *M. natalensis* which exhibited unique characters as it plotted almost at zero implying it's a generalist species. It associated equally in both fallow land and cultivated field habitats (Figure 7).

## 4 DISCUSSION

### 4.1 Small rodent species composition

This study presents the first comprehensive inventory of small rodent species in agricultural environmental setting in Uganda. Eleven small rodent species and one insectivorous mammal were recorded from both fallow land and cultivated fields. Earlier studies in the country report up to maximum of 34 small mammal species (Amatre et al., 2009; Basuta & Kasene, 1987; Clausnitzer & Kityo, 2001; Delany, 1975; Eisen et al., 2013). These report much higher species richness compared with the current study, and this was because they targeted all small mammals and their study environments (habitats) were different. For example, Eisen et al. (2013) concentrated around homesteads and within huts, where certain species almost permanently dwell such as the roof rat (*Rattus rattus*), but also migratory rodent species could be trapped in localities closer to homesteads as they search for food and escape from adverse weather conditions (Amatre et al., 2009). Particularly, some rodent species have been reported to be habitat specific, for example, *Proamys* spp. are closed forest dwellers (Basuta & Kasene, 1987) and thus could not be trapped in this study. Elsewhere in the region where studies have been conducted with similar study designs involving fallow land and maize field habitats with a capture–mark–recapture procedure, a range of between 4 and 11 species of small rodents has been reported (August, 1984; Fleming, 1975; Mares & Ernest, 1995; Makundi, Massawe, Mulungu, & Katakweba, 2010; Massawe, Rwamugira, Leir, Makundi, & Mulungu, 2006; Mulungu et al., 2013). Secondly, while the study reports eleven small rodent species, four of them which included *G. dolichurus, D. incomtus, G. kempi, and S. parvus* were very rarely encountered with less than three individuals in the whole study period. The low numbers of the later could suggest possibly unsuitable habitats for these species’ settlement, breeding, and survival (Delany, 1975; Missone, 1969). The study showed differences in species composition between fallow land and maize field habitats with higher diversity index value (0.617) for fallow land compared with cultivated fields (0.467). In Tanzania, Makundi et al. (2010) observed a similar result with a higher diversity index value in fallow land habitat compared with maize habitat. This phenomenon could be explained by land use patterns, where human activities alter habitat characteristics, which may result in a positive or negative impact on rodent communities (Hoffmann & Zeller, 2005). In this study, the authors attribute human activities including land preparation, weeding, and harvesting which are key in cultivated fields to have likely resulted into lower species richness in cultivated field habitat.

The study also showed dominance of *M. natalensis,* with over 60% contribution of the total trap catches in both habitats. This particular species is reported by several authors in the East African region as an important member of the rodent community, occurring in various habitats both disturbed and undisturbed (Hubbard, 1972; Leirs, 1995; Makundi et al., 1999, 2010; Massawe, Rwamugira, Leirs, Makundi, & Mulungu, 2005; Mulungu, 2003). The higher abundance of *M. natalensis* in cultivated fields compared with fallow land further affirm the theory that this species highly adapts to new environments and is a good colonizer of disturbed areas including cultivated agricultural fields (Leirs, 1992; Massawe et al., 2005; Makundi, Massawe, Mulungu, & Katakweba, 2010; Odhiambo, Oguge, & Leirs, 2005).

*Mus* spp. were second in abundance, which occurred equally in fallow land, and cultivated fields but more numbers in cultivated fields. This species is reported to be widely distributed across sub‐Saharan Africa where it occurs in a variety of savannah and grassland habitats (Monadjem, Taylor, Denys, & Cotterill, 2015). *Mus triton* records in this study are in total agreement with earlier taxonomic records reported in the Kenya and Tanzania (Happold, 2013; Monadjem et al., 2015; Veyrunes et al., 2004, 2005) that it occurs across the East African countries. The relatively higher numbers of *M. triton* in cultivated fields suggest that they are also good colonizers of disturbed habitats. Earlier findings by Fuller and Perrin (2001) report related results as they recovered higher numbers in a disturbed habitat that was exposed to fire. Demeke, Afework, and Gurja (2007) in Ethiopia described that *Mus* spp. were more abundant in agricultural farmland than bush habitats. *Aethomys hendei,* commonly known as bush rat, is a generalist herbivorous species and often found in woodlands although it can be found inhabiting fields that have been under cultivation (Kingdon, 1974). In the current study, more trap catches for *A. hendeii* were recovered in fallow land as opposed to cultivation field. This is typically a bush rat, which dwells in bush thickets thus the higher abundances in fallow fields signifies habitat suitability for undisturbed habitats preferably forests (Happold, 2013; Kingdon, 1974). In the current study, *Lophuromys sikapusi* was captured at relatively low numbers. An earlier study which was conducted in a national forest in the country reported relatively higher numbers compared with this study (Basuta & Kasenene, 1987). The difference can be attributed to the habitat type as this species prefers cool mist environments (Happold, 2013; Kingdon, 1974). Its preference for cooler environments was further evidenced by more trap captures in fallow than cultivated fields, which fallow exhibited microclimatic conditions (cooler undercover temperatures) that could have been enhanced by thickets of tick berry plants that were initially dominant in fallow fields. Similarly, in Tanzania, higher numbers of *Lophuromys* spp. were trapped in forest habitats particularly when vegetation was dense and humid (Makundi, Massawe, Borremans, Laudisoit, & Katakweba, 2015).

Other species captured included *A. niloticus* commonly known as African grass rat, *G. murinus* (arboreal species)*, G. kempi,* and *D. incomtus* (African Marsh rat) were recorded in relatively low numbers in the study and were mostly encountered in first year of trapping. These species were mostly captured in fallow land, a habitat which is closely related to natural forests, with relatively high weedy grasses, shrubs, trees, and form relatively dense vegetation ground cover. Such a habitat is believed to have offered favorable conditions for settlement of the above species. The results are closely related to earlier findings that reported higher numbers of *A. niloticus* during the rainy season when resources from grasses are rich with dense vegetation cover to provide shelter from predators (Massawe et al., 2005; Senzota, 1982). *G. murinus* was captured in fallow land only and encountered in the first year of trapping with no captures in the preceding years. Observations made during the study showed that vegetation cover reduced drastically in the subsequent years’ in the fallow fields due to disturbances in these fields by livestock grazing. Additionally, *G. murinus* low numbers could also be attributed to its arboreal nature as it nests on trees and routinely visits the ground thus chances of being trapped with the live Sherman traps are minimal.

### 4.2 Spatial patterns in species richness and diversity

Spatial variations in total small rodent species richness and diversity were observed, with fallow land displaying higher species richness and diversity. Similarly, spatial species turn over (β_S_) was significantly higher in fallow land habitat. The results are not surprising, as it has already been reported that habitat characteristics/patterns play a significant role in the ecology of rodents (Delany, 1975). This study further showed that while cultivated fields are less species rich, they are still very prone to infestation by rodents of different species. Specifically, *M. natalensis*, one of the notorious rodent pest species, exhibited higher rank abundance in cultivated field as opposed to fallow. This phenomenon confirms the importance of this species as an agricultural pest that calls for more attention as already reported (Makundi, Massawe, & Mulungu, 2005; Mulungu, 2003). Furthermore, Isabirye‐Basuta and Kasenene (1987) reported that the abundance and distribution of the small mammals depend mainly upon the nature and density of vegetation, which in turn influence food and shelter availability. The higher species abundances and richness in fallow fields in this study were linked to the characteristic nature of fallow land habitat which offered more vegetation for food as well as offering shelter for breeding and protection of the small rodents from possible predation as compared to cultivated fields.

Generally, habitat complexity may provide more niches that could be exploited by several species of rodents (Rosenzweig & Winakur, 1969). Niche partitioning (temporally, spatially, and trophically; Pianka, 1973) is an important factor in species co‐existence in both stable and disturbed habitats. Human activities have also been reported to significantly influence the species richness and diversity at a small scale (Massawe et al., 2005). Additionally, Getachew and Afework (2015) recovered more individuals of small rodents in bushland habitat as compared to the other habitats. This was attributed to habitat's plant composition, which included *Pterolobium stellatum, Capparis tomentosa, and Urtica simensis*, which are thorny, and prevented movement of humans and livestock, thus offering a safe environment for small mammal breeding and survival. Additionally, wild animals respond to human disturbance in the same way they respond to predation, by avoiding highly disturbed areas or underutilizing them (Beale & Monaghan, 2004; Gill, Sutherland, & Watkinson, 1996), but the strength of this response is different for different species (Gill, Norris, & Sutherland, 2001). In this study, species richness and abundance were high in fallow land habitat, which could possibly be due to the low levels of human activities/disturbance as compared to cultivated field.

### 4.3 Temporal patterns in total species richness and abundance

In the current study, temporal variations were an important factor that influenced the species richness and relative abundance of the species across the fields. The monthly year to year changes in small rodent species richness and abundance were also obvious, with higher richness and abundance in the first year of trapping compared with the proceeding years of trapping. There were significant variations in monthly rodent species richness and abundances over the two and half years of the study period with generally higher richness and abundance in the months of June, July, and August in 2016 and March and April in 2018 trapping. These results are similar with earlier studies by Makundi et al., (2010) and Mulungu (2003), when they recovered more species and higher trap catches in the first year of the study. The monthly changes in small rodent abundance reported here only affirm earlier theories that suggest that rodent populations are highly dynamic and are driven by several environmental factors, but more particularly by rainfall, which influences vegetation and human activities (Leirs, 1992). It was noted that vegetation cover and human activities changed with months, and this is believed to have played a role in regulating rodent populations in both habitats. For example, due to constant human activities in cultivated fields, the rodent populations fluctuated more highly as opposed to fallow land where it was observed to have had minimal human interaction. Similar observations are reported by Addisu and Bekele (2013) who report that crop harvesting and grazing were perhaps the considerable factors for the reduction in rodent's abundance in maize fields during the dry season in their study in Ethiopia. Specifically, increased animal grazing has been widely shown to affect rodent species composition and abundance (Cao et al., 2016; La Morgia, Balbo, Memoli, & Isaia, 2015; Yihune & Bekele, 2012). Additionally, habitat fragmentation and anthropogenic activity can make areas inviable for certain fauna and can therefore alter their distribution (Markovchick‐Nicholls et al., 2008).

Nevertheless, several publications report temporal rodent abundances in terms of months and years various explanations are given. For example, Mulungu et al. (2013) reported lower rodent abundances but with more female individuals breeding during the rainy season. Similarly, Massawe, Makundi, Mulungu, Katakweba, and Shayo 2012report breeding patterns of some rodent species in Central Tanzania to be seasonal and correlated well with rainfall patterns. Other studies on ecology of rodents in East Africa have associated population dynamics with the indirect influence of rainfall on reproduction patterns and habitat characteristics, including vegetation structure and cover (Delany, 1972; Leirs, 1992; Leirs, Verheyen, Michiels, Verhagen, & Stuyck, 1989; Makundi et al., 2005; Makundi, Massawe, & Mulungu, 2006; Taylor & Green, 1976; Telford, 1989). Precipitation has been reported to result into increased primary vegetation production, which in turn leads to increased rodent abundance (Gage & Kosoy, 2005). The temporal differences observed in the current study are likely attributed to several factors already reported on in earlier studies but were not quantitatively analyzed in this study, which include among others, vegetation ground cover, quality food supply, and human activities which are all governed by rainfall. Already, existing theories show that human activity can have negative impacts on many wildlife species, leading to changes in distribution (moving away from human activity), abundance, and activity patterns (Griffiths & Van Schaik, 1993). This type of scenario indeed was observed in fallow fields where species richness and abundance were high in the first year of the study but declined with time due to increased pressure as a result of human activities on this reserved piece of land. Human activities such as animal grazing have thus been observed to have an impact on rodent species distribution and abundance and can be used as a means of modifying environment as a rodent management technique in a localized setting (La Morgia et al., 2015).

Consequently, although there were no clear trends in the population dynamics from one year to another, a less similar pattern of increased rodent abundance in the second part of year from May to November was noted throughout the two and half years of the study.

### 4.4 Synthesis and applications

Long‐term studies that provide description of rodent species composition and community structure in agriculture settings contribute to state of the pest management reports, environmental risk assessments, and offer options for harmonizing the benefits of rodents in the ecosystem and protection from pest damage. Currently, most descriptions focus on rodents as forest dwellers as indicators of habitat quality and, as vectors of human and livestock diseases (Clausnitzer, Church, & Hutterer, 2003; Eisen et al., 2013). On the contrary, this study focused on understanding rodents as pests in a crop farming system to establish the common species and how they are distributed between cultivated fields and fallow land in such an agricultural setting.

Therefore, an understanding of rodent species composition for a given locality is particularly valuable; for conservation and management purposes. Gorvnment agencies responsible wild life conservation and pest control can utilize the information for approprite decision making for conservation and application of appropriate control measures on pestivorous species respectively. Our approach identifies the most abundant species in cropped fields and relates with other studies in the region on potential impacts these species can have on crops in an agricultural system.

## CONFLICT OF INTEREST

None declared.

## AUTHORS' CONTRIBUTION

Mayamba. A, Mulungu. L. S, Makundi. R.H, and Massawe. A conceived the ideas and designed methodology. All authors contributed to data collation. Mayamba A, Mulungu. L.S, and Isabirye. B analyzed the data and led the writing of the manuscript. All authors critically reviewed the drafts and gave final approval for publication.

## Data Availability

We the authors of this manuscript have collectively agreed to have the data used in the results section to publicly avail that information to a public domain Dryad once this paper has been accepted for publication under the journal of Ecology and Evolution. https://doi.org/10.5061/dryad.qv9471s.
